# A De Novo Designed Trimeric Metalloprotein as a Ni_p_ Model of the Acetyl-CoA Synthase

**DOI:** 10.3390/ijms241210317

**Published:** 2023-06-19

**Authors:** Dhanashree Selvan, Saumen Chakraborty

**Affiliations:** Department of Chemistry and Biochemistry, University of Mississippi, Coulter Hall, Oxford, MS 38677, USA

**Keywords:** carbon cycle, acetyl-CoA synthase, de novo proteins, metal-carbonyl, nickel, enzyme models

## Abstract

We present a Ni_p_ site model of acetyl coenzyme-A synthase (ACS) within a de novo-designed trimer peptide that self-assembles to produce a homoleptic Ni(Cys)_3_ binding motif. Spectroscopic and kinetic studies of ligand binding demonstrate that Ni binding stabilizes the peptide assembly and produces a terminal Ni^I^-CO complex. When the CO-bound state is reacted with a methyl donor, a new species is quickly produced with new spectral features. While the metal-bound CO is albeit unactivated, the presence of the methyl donor produces an activated metal-CO complex. Selective outer sphere steric modifications demonstrate that the physical properties of the ligand-bound states are altered differently depending on the location of the steric modification above or below the Ni site.

## 1. Introduction

In early Earth, life prevailed under anaerobic conditions. In carbon fixation pathways, many anaerobic organisms grow autotrophically using CO as their sole source of energy. Perhaps more surprisingly, at very low concentrations CO is implicated to produce clinical benefits in cardiovascular diseases, inflammatory disorders, and organ transplantation [1]. The beneficial effect of CO has been linked to heme oxygenase-1 activity since this enzyme plays an important role in stress response and injury. In addition to the biological importance of CO, it is also a critical component in many industrial processes. For example, a metal-CO species is utilized in the water-gas shift reaction, the Monsanto acetic acid synthesis process, the Reppe process for CO insertion to unsaturated compounds, and the Fisher-Tropsch process. 

**Figure 1:**
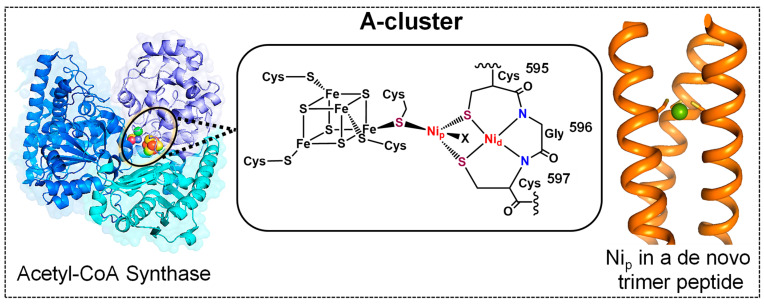
The structure of ACS and the A-cluster. A PyMol model of Ni_p_ analog Ni-CS1 is shown on the right (orange cartoon), with the Ni as a green sphere.

Carbon monoxide dehydrogenase (CODH) and acetyl coenzyme-A (CoA) synthase (ACS) are closely associated enzymes that play a vital role in the Wood-Ljungdahl pathway of the global carbon cycle [2,3]. The CODH/ACS enzyme is a 310 kDa α2β2 heterotetramer that catalyzes two important reactions. The C cluster in the CODH subunit (β) converts CO_2_ to CO, whereas the A-cluster in the ACS subunit (α) couples CO and a -CH_3_ group to produce an acetyl-complex, that is thiolyzed by CoA to make acetyl-CoA, which is a vital carbon source in these organisms [4]. The crystal structure of CODH/ACS from acetogenic bacteria *Moorella thermoacetica* showed that the A-cluster is made of a [Fe_4_S_4_] cubane linked to a Cu-Ni binuclear site [4]. The subsequent structure showed the presence of an open and a closed conformation of the α subunit. The closed form contained one Zn and one Ni atom at the A-cluster, whereas the open conformation showed two Ni atoms [5]. Based on this structure, spectroscopy, and the fact the enzyme activity correlated with Ni and not with Cu, it has since been established that the active A-cluster (Figure 1) consists of a [Fe_4_S_4_]-linked to two Ni atoms [5]. The Ni sites are recognized based on their proximity from the [Fe_4_S_4_] cluster, e.g., the proximal (Ni_p_) and distal (Ni_d_). Ni_p_ is the redox-active site where substrate binding and activation occur. The Ni_d_, on the contrary, remains a redox-inactive spectator during catalysis. Ni_p_ consists of a Ni(Cys)_3_ binding motif with Cys^595^ and Cys^597^ plus a bridging cysteine (Cys^509^) from the [Fe_4_S_4_] cluster in an apparent T-shaped geometry. The Ni_d_ site is in a square planar environment where Cys^595^, Cys^597,^ and the nitrogen atoms from the amide backbone of Cys^595^-Gly^596^-Cys^597^ tripeptide act as the ligands. The square planar Ni geometry is proposed to be resistant to accommodate oxidation state-dependent structural changes of Ni, which is a reason for the redox inactive nature of the Ni_d_ site.

Several synthetic models of both the Ni_p_ and Ni_d_ sites have been reported [6,7,8,9,10,11,12]. Rauchfuss and coworkers used a NiS_2_N_2_ complex to make Cu-Ni and Ni-Ni complexes as first-generation models of ACS with υ(CO) bands at 1948 cm^−1^ and 1866 cm^−1^ [6]. Mascharak and coworkers reported several model systems [8,9] as synthons of Ni_d_, Ni-Cu, and Ni-Ni dinuclear complexes. The Ni_p_ site in the Ni-Ni dimer bound CO in its reduced form, while the Ni_d_ site did not, confirming that Ni_p_ is the catalytically active center. In a separate study [9], the formation of trinuclear Ni-Cu-Ni and Ni-Ni-Ni complexes was demonstrated. The trinuclear Ni-Cu-Ni species with two NiN_2_S_2_ units linked to Cu^I^ through sulfur bridging was not reactive towards CO under various conditions. This led to the synthesis of a trinuclear Ni species with three coplanar Ni centers where the central Ni (Ni_C_) served as a structural model of the Ni_p_ site. In the reduced form this complex displayed a strong EPR signal with g values 2.33 and 2.09 and was also shown to bind CO terminally with a υ(CO) of 1960 cm^−1^. Tatsumi and coworkers reported dinuclear Ni complexes [10,11] capable of CO insertion to a Ni-CH_3_ bond. A -Cys-Gly-Cys- tripeptide as a Ni_d_ site model was reported by Riordan and coworkers [13], which showed that Ni-binding can occur without a large protein structure, yet representing a synthetic analog of the Ni_d_ site [13]. The K_2_[Ni(CGC)] peptide was used as a precursor to building high-nuclearity Ni complexes.

Holm and coworkers designed a helix-loop-helix 63-mer peptide [14,15] where the loop sequence from ferredoxin was used to incorporate [Fe_4_S_4_] clusters with one of the Cys acting as a bridging ligand. An N_3_S site with 3His/1Cys ligands, and an N_2_S_2_ site with 2His/2Cys ligands were introduced by placing these motifs in the proximity of a synthetic [Fe_4_S_4_] cluster incorporated via Cys ligands from the peptide [14,15]. Shafaat and coworkers designed azurin-based (Az) models featuring the 2His/1Cys binding motif of Az [16]. Under reducing conditions, NiAz forms a trigonal planar Ni^I^ site, similar to the reduced state in ACS. Since NiAz lacks a well-defined substrate binding pocket, the axial Met121 was replaced by Ala to allow substrate access. The M121A-Ni^I^Az binds CO, producing a Ni^I^-CO specie that was characterized using UV-vis, FTIR, EPR, and theoretical methods. The NiAz system is accessible to Ni^I^, Ni^II^, and Ni^III^ oxidation states, which are relevant oxidation states proposed in the catalytic cycle of the ACS. In EPR experiments a large degree of spin-orbit coupling was observed, suggesting a Ni-species with an unpaired electron in d_x2-y2_ or d_xy_ orbital. The observed g values were supported with DFT calculations as well [17,18]. The reactivity of M121A-Az was tested using various methyl donors, producing a Ni^III^-CH_3_ species that further reacted with CO. 

**Figure 2:**
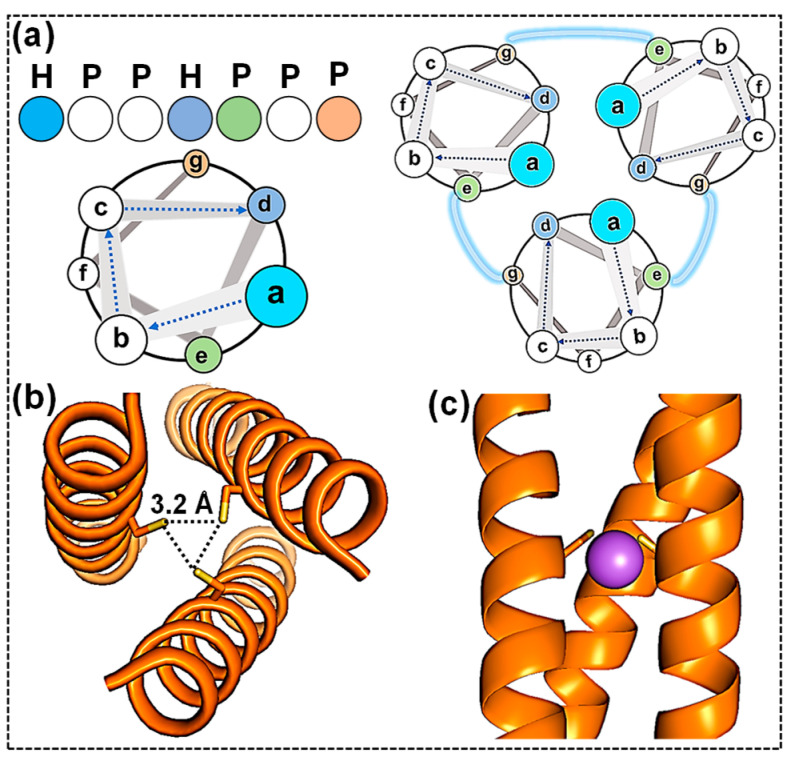
(**a**) Helical wheel diagrams showing the assembly of de novo peptides into a trimer as driven by the knobs-into-hole packing of the hydrophobic *a* and *d* residues. X-ray structures of (**b**) apo (PDB 3LJM) and (**c**) As^III^-(CSL9C)_3_ (PDB 2JGO). The major Cys orientation is shown in (**b**).

De novo protein design is a complementary approach that helps delineate the sequence-to-structure relationships of complex enzymes [19]. De novo protein design allows the creation of structural and functional analogs of native metalloenzymes that can be studied in aqueous conditions with the metal site embedded in a polypeptide matrix. Here, we have used a de novo-designed trimer peptide (Figure 2a) [20,21], as a scaffold to mimic the Ni_p_ (Cys)_3_ site of ACS. To the best of our knowledge, this is the first example of a NiS_3_-type active site model of the Ni_p_ site of the A-cluster in a de novo-designed protein scaffold.

## 2. Results and Discussion

### 2.1. Scaffold Choice to Host the Ni(Cys)_3_ Site

De novo peptides designed according to the heptad repeat [*abcdefg*]_n_ self-assemble in solution where the oligomeric state is determined according to knobs-into-holes packing of the hydrophobic *a* and *d* residues at the core of the assembly (Figure 2b) [22,23]. To host the NiS_3_ site, analogous to the Ni_p_, we have chosen the de novo designed peptide Coil Ser (CSL9C) [20] that was previously shown to bind heavy metals. In the X-ray structure of As^III^-(CSL9C)_3_, the As^III^ is shown to be located in a plane below the coordinating Cys 9 layer (Figure 2c) [21]. We hypothesized that the thiol-rich pocket of CSL9C can serve as a scaffold to host Ni^II^ in a symmetric coordination environment. We introduced a point mutation H28Q to avoid competitive Ni binding with this external solvent exposed His residue. This mutant is designated hereafter as CS1 and used in this study.

### 2.2. Ni Binding

**Figure 3:**
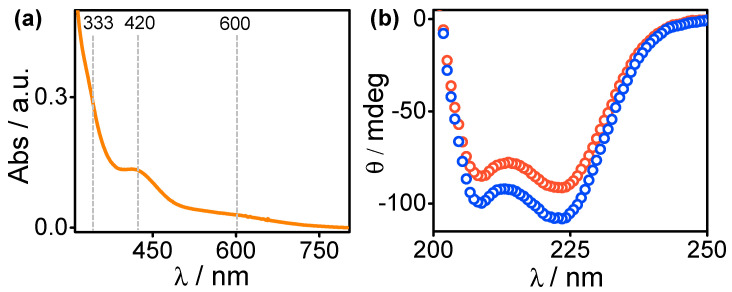
(**a**) The UV-vis spectrum of 1.2 mM Ni^II^-CS1. (**b**) CD spectra of apo-CS1 (red) depicting the α-helical nature of the peptide and Ni^II^-CS1 (blue) showing an increase in helicity upon Ni binding. CD data were collected at ~33 μM trimer concentration. All data were collected at pH 8.5.

**Figure 4:**
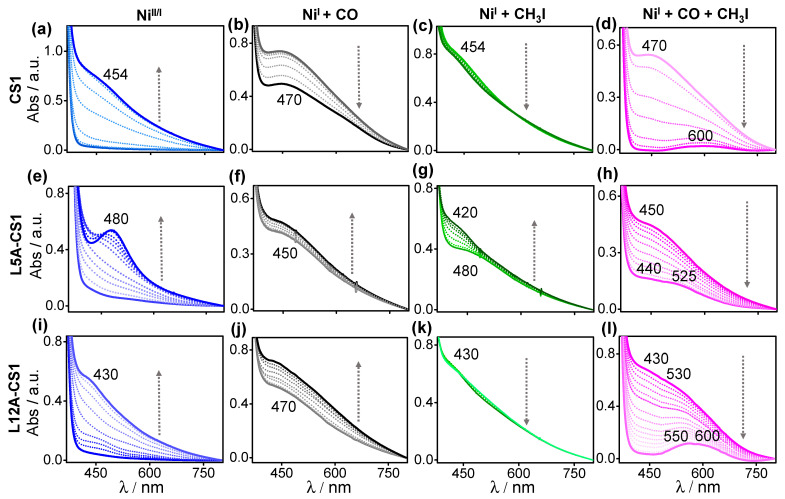
Ligand binding kinetics for CS1, L5A-CS1, and L12A-CS1. (**a**,**e**,**i**) Reduction of Ni^II^-peptides using dithionite; (**b**,**f**,**j**) CO binding to Ni^I^-peptides; (**c**,**g**,**k**) Kinetics of CH_3_I binding to Ni^I^-peptides, and (**d**,**h**,**l**) show the kinetics of Ni^I^-peptide-CO with the methyl donor. Samples contained 480 μM peptide trimers in 50 mM Tris pH 8.5, and 120 mM KCl. Sample preparation and data collection were performed anaerobically. CO-saturated buffer was added to Ni^I^-peptides to monitor the CO binding. Ni^I^-peptides were prepared with 50-fold excess dithionite. CH_3_I was used in 50-fold excess.

**Figure 5:**
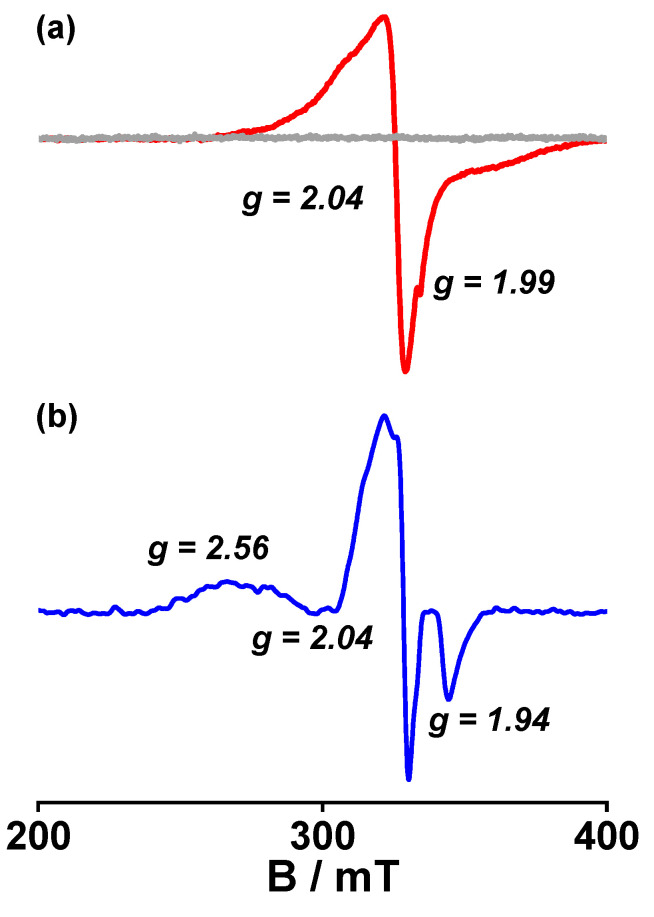
The CW X-band EPR spectra of (**a**) Ni^I^-CS1 (red), the EPR silent Ni^II^-CS1 control sample (gray); and (**b**) Ni^I^-CS1-CO. Spectral changes in the CO-bound state are observed compared to Ni^I^-peptide. T = 15 K. Peptide concentration is 1 mM trimer. pH = 8.5.

Ni binding to CS1 was studied using UV-vis and circular dichroism (CD) spectroscopy. The UV-vis spectrum (Figure 3a) of ~1.2 mM CS1 treated with Ni^II^ shows a prominent peak at ~420 nm (ε = 111 M^−1^cm^−1^), plus a shoulder at ~333 nm (ε = 290 M^−1^cm^−1^) and a broad feature at ~600 nm (ε = 25 M^−1^cm^−1^). Metal binding was further verified by CD, where an increase in the α-helical character was observed upon Ni^II^ addition (Figure 3b, blue) to apo CS1 (Figure 3b, red). Combined, these data demonstrate Ni^II^ binding to the peptide [24,25]. To characterize Ni^I^-states, Ni^II^-CS1 was treated with excess sodium dithionite, and the reduction kinetics was monitored (Figure 4a). In these experiments, a lower peptide concentration of 480 μM was used to avoid detector saturation. At this concentration, the Ni^II^ features are weak and not visible due to the low extinction coefficient of the absorption bands. Upon reduction, which occurred within ~15 min, a broad peak at ~454 nm appears. From the analysis of the kinetic data, the initial rate of formation of Ni^I^-CS1 was calculated to be 3.65 × 10^−4^ s^−1^ (Table 1) assuming a pseudo-first-order reaction. The electron paramagnetic resonance (EPR) spectrum of Ni^II^-CS1 is featureless (Figure 5a, gray), as expected for a d^8^ Ni^II^ system. The dithionite-reduced sample showed an anisotropic EPR signal with g values at ~2.04 and ~1.99, corresponding to the formation of S = 1/2 Ni^I^ (Figure 5a, red) species. Combined, these data show that Ni^II^-CS1 is reducible to Ni^I^-CS1.

### 2.3. Characteristics of Ligand-Bound States

**Scheme 1:**
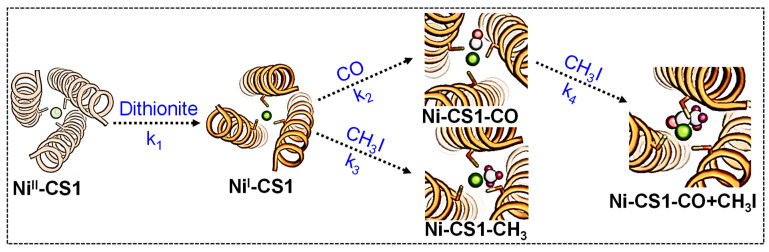
Ni^II^ reduction and ligand binding kinetics to the reduced state.

Binding of CO and -CH_3_ to Ni^I^-CS1 was monitored by UV-vis kinetics (Scheme 1). Upon addition of a CO-saturated buffer solution to Ni^I^-CS1, a red-shifted transition from 454 nm to 470 nm was observed within 500 ms, which suggests that CO binds to Ni^I^-CS1 (Figure 4b). This observation resonated with the fact that Ni^I^ is the active form that binds CO due to favorable back-bonding from Ni^I^ to CO. The pseudo-first-order rate constant for Ni^I^-CO formation was found to be 2.74 × 10^−4^ s^−1^ (Table 1). Next, we monitored the reaction of Ni^I^-CS1 with CH_3_I as a model methyl donor. As shown in Figure 4c, only minute changes were observed in the UV kinetics. Finally, to assess whether the CO-bound state reacts with -CH_3_I, we treated Ni^I^-CS1-CO with CH_3_I. As shown in Figure 4d, the 470 nm peak of Ni^I^-CS1-CO decayed upon reaction with CH_3_I, maturing with a new feature generated at ~600 nm (k_4_~1.10 × 10^−3^ s^−1^; Table 1).

**Figure 6:**
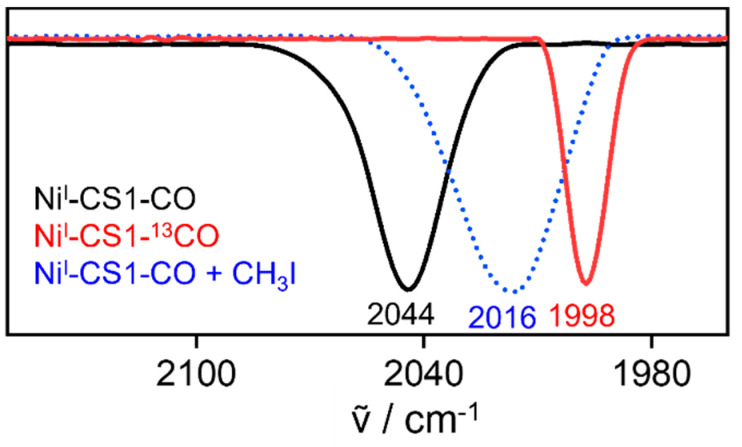
FTIR spectra of Ni^I^-CS1-CO (black), Ni^I^-CS1-^13^CO (red), and Ni^I^-CS1-CO plus CH_3_I (dotted blue). Samples were prepared anaerobically and measured in FTIR cells with CaF_2_ windows.

We further studied ligand binding to Ni^I^-CS1 using FTIR. In the CO-bound form of Ni^I^-CS1, a peak at ~2044 cm^−1^ is observed (Figure 6), representative of a terminal Ni-CO species [8,26]. The observed peak is at higher energy than the 1996 cm^−1^ band assigned to the CO-bound A-cluster of ACS [26].

Ragsdale and coworkers observed multiple bands in the range of 2100–1900 cm^−1^ for a terminal carbonyl-bound C cluster of CODH [26]. The Ni^I^-CO stretching frequencies in a model Ni^I^-CO complex where Ni is coordinated to three thioethers has a υ(CO) of 1999 cm^−1^ while the Ni^I^ complex with two thiolate and two nitrogen ligands show υ(CO) of 2040 cm^−1^ [26,27,28]. The reaction of a binuclear Ni-Ni complex with CO resulted in a terminal Ni^I^-CO (N_2_S_2_ coordination) band at 2044 cm^−1^ [8]. The M121A-Az model (2His/1Cys coordination) displayed a stretching frequency of 1976 cm^−1^ [17] in its CO-bound state. These results show that the ligand and coordination environment strongly influences the nature of M-CO species produced in model complexes and biomolecular models. To further evaluate CO binding, we probed the isotope effect by characterizing the ^13^CO-bound form of Ni^I^-CS1 (Figure 6 red). In this case, an FTIR band at 1998 cm^−1^, which is similar to the calculated isotopic band of 1999 cm^−1^, is observed with a Δυ (^12^CO−^13^CO) of 46 cm^−1^. This frequency difference is similar to 44 cm^−1^ observed in the Ni-azurin model system as well [17]. The Ni^I^-CS1-CO has a rhombic EPR spectrum with g values of ~2.56, 2.04, and 1.94 (Figure 5b). The NiS_3_-CO type molecule, reported by Riordan and coworkers, showed a rhombic EPR signal with a significant axial character, having g values of 2.64, 2.02, and 1.95 [29]. In CO-bound Ni^I^-Az, an EPR signal with g values of 2.37, 2.17, and 2.00 were observed, which is similar to that of the M121A-Az mutant which has g values of 2.30, 2.20, and 2.00 [17]. The NiFeC signal in CO-bound A-cluster (A_red_-CO) has g values of 2.08 and 2.03 [30]. The FTIR spectrum after methyl addition to Ni^I^-CS1-CO has a frequency of ~2016 cm^−1^ (Figure 6 blue), which is lower than the CO-bound state alone, indicating a reduced bond order of CO upon -CH_3_ binding.

### 2.4. The Effect of Outer Sphere Steric on Ligand Binding

**Figure 7:**
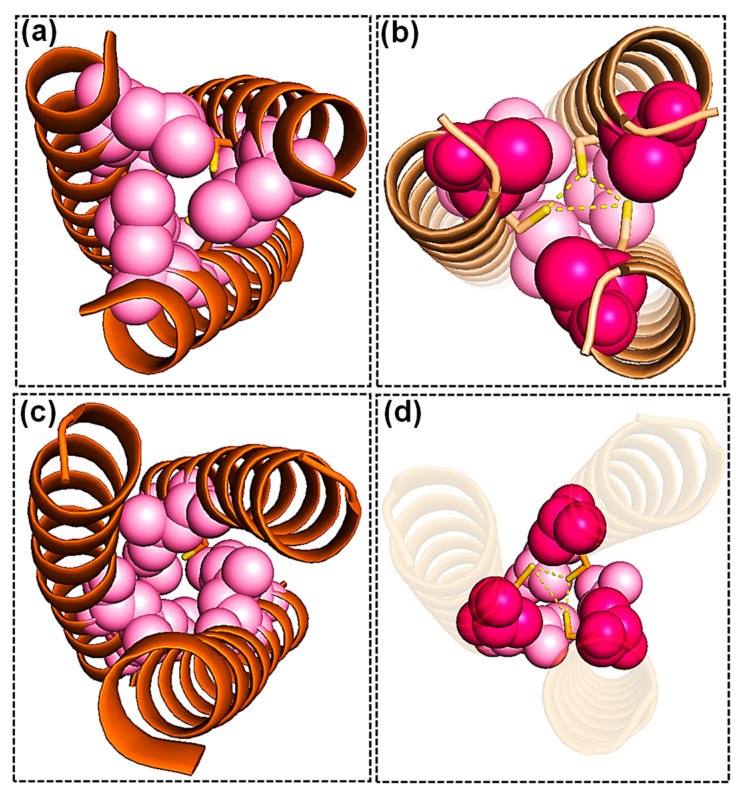
Space-filling models showing the packing of the 5th layer Leu from the N-terminus of CS1 (**a**) and after mutation to Ala (**b**). The corresponding view of the 12th layer Leu from the C-terminus (**c**), and the Ala mutant (**d**). Here, the Leu layers are shown in light pink and the Ala layers are shown in hot pink. Cys are shown as orange/yellow sticks.

We hypothesized that creating more space above or below the plane of Ni could provide easier access to the ligands. To assess the effect of sterics on ligand binding to the NiS_3_ site, we prepared two mutants L5A-CS1 and L12A-CS1 by mutating the Leu at the 5th or 12th position to Ala. The first mutation is at the top of the Ni site toward the N-termini, while the second mutation is at the bottom of the Ni site towards the C-termini. These mutants are also used to evaluate if any preference for ligand binding towards the top or the bottom of the Ni site exists. Models generated in CC Builder [31] show that these mutations indeed open up space above and below the Cys plane (Figure 7). In the mutant models, the Cys residues are found to be located at a distance of 2.5Å, 3.3Å, and 4.4Å from each other. Such orientation is different from the X-ray structure of As^III^-CSL9C [21] where the Cys residues are symmetrically oriented from one another due to the binding of As^III^. In apo CSL9C, the Cys are oriented either towards the interior or towards the helical interface, as governed by the rotameric preference of Cys [20]. The S atoms of Cys in the mutants are asymmetrically oriented. The UV-vis kinetic studies of ligand binding to the mutants were performed similarly to Ni^II^-CS1.

The reduction of Ni^II^ to Ni^I^ is accompanied by the appearance of prominent bands at 480 nm in Ni^II^-L5A-CS1 (Figure 4e) and at 430 nm in Ni^II^-L12A-CS1 (Figure 4i). The reduction occurred with similar initial rate constants of 1.28 × 10^−3^ s^−1^ and 1.22 × 10^−3^ s^−1^ (Table 1) for L5A-CS1 and L12A-CS1, respectively. These rates were ~3.5 times faster than the reduction of CS1. Better access to the reductant likely improved the rate of reduction in the mutants compared to the parent CS1 peptide. Upon CO binding to Ni^I^-L5A-CS1, a peak at 450 nm was observed (Figure 4f), blue-shifted from 480 nm without CO. The Ni^I^-L12A-CS1 displayed a red shift in peak from 430 nm to 470 nm (Figure 4j) with CO, showing a similar trend to the binding of CO in Ni^I^-CS1. In the presence of CH_3_I, Ni^I^-L5A-CS1 depicted a very clear blue shift from 480 nm to 420 nm (Figure 4g), while Ni^I^-L12A-CS1 showed no noticeable changes (Figure 4k). When the CO-bound mutants were treated with CH_3_I, the reaction kinetics appeared to be slower than the Ni^I^-CS1. We attribute this observation to the fast coupling of CO and -CH_3_ ligands in Ni^I^-CS1, which is likely attributed to a local difference in the coordination environment of the Ni center in CS1 compared to the mutants. Upon -CH_3_ binding, the 450 nm peak in Ni^I^-L5A-CS1-CO decayed to 440 nm and 525 nm (Figure 4h), while for Ni^I^-L12A-CS1-CO, we observed that the peaks at 430 and 530 nm decayed to a broad feature with absorbance at 550 nm and 600 nm (Figure 4l). The FTIR data for the mutants in their ligand-bound states (Figure S1) are similar to the CS1 peptide, which suggests a similar chemical nature of the resultant species.

From kinetic studies of mutants, we find that the mutation above or below the plane of the active site has increased the rate of reduction of Ni^II/I^ while the reaction of CO and CH_3_I to Ni^I^ did not affect the rate. In the presence of both CO and -CH_3_ ligands the reaction kinetics was slower than the parent peptide. An interesting observation from the UV-vis plots is that the features in CS1 and L12A-CS1 are similar in all the ligand-bound states. On the contrary, there are notable changes in the UV-vis features of L5A-CS1 compared to the parent peptide and the other mutant. This observation suggests that the hydrophobic residues above the Ni-CS1 center play a major role in controlling the reaction of ligands with the active site.

## 3. Materials and Methods

### 3.1. Experimental Procedures

CO (99.997%) with O_2_ content <3 ppm and ^13^CO (99.99%) with <15 ppm O_2_ content were purchased from nexAir (Memphis, TN, USA) and Sigma-Aldrich (St. Louis, MO, USA), respectively. The trace amounts of oxygen were removed by passing the gases through a 6300 series oxygen trap from nexAir. CH_3_I (99%) was purchased from Sigma-Aldrich. Ultrapure Tris (VWR, Randor, PA, USA), KCl, NaOH (Fisher, Hampton, NH, USA), NiSO_4_.6H_2_O (Sigma-Aldrich), and Sodium hydrosulfite (Alfa Aesar, Haverhill, MA, USA) were used as received without further purification.

### 3.2. General Procedures

All buffers were chelexed (Sigma-Aldrich) overnight, pH adjusted, and filtered before use. All the glassware was treated using EDTA and 10% nitric acid and autoclaved.

### 3.3. Peptide Synthesis

The solid-state peptide synthesis was performed using a CEM Liberty Blue (Matthews, NC, USA) automated microwave peptide synthesizer as per our earlier reports [24,32].

### 3.4. Mass Spectrometry

The mass of the crude and the purified peptide was determined using MALDI-MS (Bruker, Billerica, MA, USA) by preparing a 1:2 dilution of the peptide and the matrix (Sinapinic acid; Sigma-Aldrich) solution. These solutions were dried on a stainless steel MALDI plate before collecting the data. A 10 mg/mL solution of the matrix was prepared in 50:50 water/acetonitrile solution with 0.1% trifluoracetic acid (TFA, Advanced ChemTech, Louisville, KY, USA).

### 3.5. DTNB Assay

The thiol quantification was done using Ellman’s reagent 5,5′-dithiobis-(2-nitrobenzoic acid) (DTNB) from Acros Organics (New Jersey, USA). The number of free Cysteines was determined to be ~2.8.

### 3.6. Sample Preparations

All sample preparations were done inside a glove box (MBraun, Stratham, NH, USA) having <0.5 ppm O_2_. The buffers were degassed thoroughly for 5–6 h, transferred into the box, and stirred overnight openly in the box atmosphere to remove any remaining O_2_ before using them for experiments. The apo peptides were dissolved in 50 mM Tris pH 8.5, and 120 mM KCl, and their concentration was determined using ε_280_ = 5500 M^−1^ cm^−1^. Metalation was performed with 1.2 equivalents of NiSO_4_.6H_2_O with 0.5 equivalent at a time and stirring for ~15 min in-between additions. The Ni^II^-peptides were treated with 50-fold excess dithionite to make Ni^I^-CS1. Excess dithionite was removed by a PD10 column. The CO-bound species were prepared by purging CO into Ni^I^-CS1 solutions for ~50–60 min. The -CH_3_ derivatives were prepared by adding 50-fold excess methanolic CH_3_I solutions to precursors.

### 3.7. UV-Vis Spectroscopy

UV-vis kinetics experiments were performed on an Agilent Cary 8454 instrument using an anaerobic cuvette (Starna Cells). The kinetics of Ni^II^ to Ni^I^ conversion was recorded by adding degassed dithionite solutions (excess) to peptides prepared inside the box and recording the change in absorbance every 0.5 s. In separate experiments, to probe CO binding, we saturated the 50 mM Tris pH 8.5, and 120 mM KCl buffer solutions with CO for ~1 h. This CO-saturated buffer was added to dithionite-reduced Ni^I^-CS1 solutions and the kinetics was monitored. Similarly, -CH_3_ binding kinetics were studied by adding CH_3_I to degassed methanol and then using this solution with the respective precursor peptide samples. Kinetics for L5A-CS1 and L12A-CS1 mutants were performed using a similar procedure.

### 3.8. CD Spectroscopy

CD data were collected on a JASCO spectrometer (Easton, PA, USA) using a 1 cm pathlength quartz cuvette. Samples contained ~30–40 µM trimer peptides in 5 mM Tris pH 8.5 with or without Ni^II^.

### 3.9. FTIR Spectroscopy

FTIR data were collected using a PIKE (Madison, WI, USA) demountable liquid cell holder with CaF_2_ windows in a Tensor FTIR instrument (Billerica, MA, USA). We used a gas-tight syringe to transfer the samples (~60 uL) into the liquid cell through luer locks and capped them inside the anaerobic chamber. Before each experiment, background scans were collected with the buffer followed by the sample spectrum of up to 1000 scans with a resolution of 2 cm^−1^ in the range of 4000 to 1000 cm^−1^. The ^13^C labeled samples were also prepared similarly.

### 3.10. EPR Spectroscopy

EPR samples contained ~1 mM peptides in 50 mM Tris pH 8.5, and 120 mM KCl plus 25% glycerol. All samples were prepared in septa-capped vials under strict anaerobic conditions, which were transferred to EPR tubes and immediately frozen in liquid nitrogen. The samples with CO and CH_3_I were frozen very slowly to minimize the chances of breaking the EPR tubes. The sample spectra were collected at 15K using a Bruker EMXPlus X-band Continuous Wave EPR instrument (Billerica, MA, USA).

## 4. Conclusions

We have employed a de novo-designed trimer peptide to incorporate a Ni(Cys)_3_ motif to produce an analog of the Ni_p_ site of ACS within a biomolecular scaffold. Metal binding is shown to stabilize the peptide assembly. From the kinetics studies, the fastest rate is obtained when Ni^I^-CS1-CO reacts with CH_3_I. EPR data of Ni^I^-CS1 and its CO-bound state show anisotropic EPR signals where the reaction of CO with the peptide leads to spectral changes. FTIR spectra of the CO-bound state and the isotope effect with ^13^CO are characteristic of a terminal Ni^I^-CO species with an unactivated CO moiety. CH_3_I appears to have a low reactivity to Ni^I^-CS1. However, in the CO-bound state, the reaction of CH_3_I is quick and leads to the formation of a species with distinct UV-vis spectra. CH_3_I addition produces an activated Ni-CO species as evidenced by a decrease in FTIR frequency compared to Ni-CS1-CO alone. Variants lining the top and bottom layers of the Ni site show increased kinetics for the reduction of the Ni site; however, the behavior with the ligands remains unchanged. Interestingly, the spectral signatures of the parent peptide and the variant that has decreased steric below the Ni site towards the C-termini are quite similar to each other. In contrast, the variant with more space above the Ni site has different spectral features than the above two peptides. It appears that altered steric interactions affect the ligand binding properties of the Ni site differentially in these variants.

## Figures and Tables

**Table 1:** Results from the kinetics of reduction and ligand (CO, CH_3_I) binding to Ni^I^-CS1, Ni^I^-L5A-CS1, and Ni^I^-L12A-CS1.

	k_1_ (s^−1^)	k_2_ (s^−1^)	k_3_ (s^−1^)	k_4_ (s^−1^)
**CS1**	3.65 × 10^−4^	2.74 × 10^−4^	6.81 × 10^−5^	1.10 × 10^−3^
**L5A-CS1**	1.28 × 10^−3^	5.10 × 10^−4^	9.86 × 10^−5^	5.22 × 10^−4^
**L12A-CS1**	1.22 × 10^−3^	3.55 × 10^−4^	9.16 × 10^−5^	4.69 × 10^−4^

## Data Availability

Data is available upon request from the authors.

## Supplementary Materials

The following supporting information can be downloaded at: https://www.mdpi.com/article/10.3390/ijms241210317/s1, Figure S1: FTIR data of ligand-bound states of the mutants.
