# Supplementary material for: A De Novo Designed Trimeric Metalloprotein as a Nip Model of the Acetyl-CoA Synthase
Source: Int J Mol Sci. 2023 Jun 19;24(12):10317. doi: 10.3390/ijms241210317 (PMC10299331; doi:10.3390/ijms241210317)
Supplement: Supplementary file 1 [file ijms-24-10317-s001.zip › ijms-2349769-supplementary.pdf]

Supporting Information

A De Novo Designed Trimeric Metalloprotein as a Ni_p Model of the Acetyl-CoA Synthase

Dhanashree Selvan, Saumen Chakraborty *

Department of Chemistry and Biochemistry, University of Mississippi, Coulter Hall, Oxford MS, USA
Email: saumenc@olemiss.edu

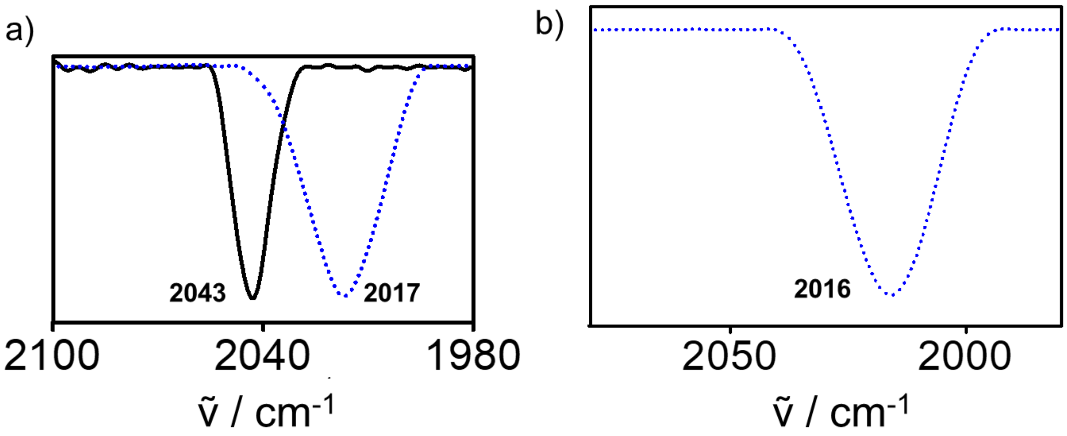

Figure S1. a) FTIR spectra of Ni^{I}-L5A-CS1 with CO (black trace) and with CO + CH_3I (dotted blue trace); b) Ni^{I}-L12A-CS1 with CO and CH_3I (dotted blue). The corresponding spectrum for CO-bound Ni^{I}-L12A could not be obtained.
